# Immunoadsorption-Based Desensitization With Column Reuse in ABO-Incompatible Living Donor Renal Transplantation

**DOI:** 10.7759/cureus.99538

**Published:** 2025-12-18

**Authors:** Pranjal Kashiv, Mohit Kurundwadkar, Prasad Gurjar, Mohan Patel, Shubham Dubey, Kapil N Sejpal, Priyanka Tolani, Amit S Pasari, Manish Balwani, Vivek Kute

**Affiliations:** 1 Nephrology, Jawaharlal Nehru Medical College, Wardha, IND; 2 Nephrology, Apollo Hospital, Nashik, IND; 3 Internal Medicine, Jawaharlal Nehru Medical College, Wardha, IND; 4 Nephrology, Saraswati Kidney Care Center, Nagpur, IND; 5 Nephrology, Institute of Kidney Diseases and Research Centre, Ahmedabad, IND

**Keywords:** aboi transplantation, column reuse, desensitization, immunoadsorption, plasmapheresis

## Abstract

Introduction: ABO-incompatible kidney transplantation (ABOi-KT) has become a viable solution to expand access to living donor transplantation, particularly in countries where deceased donor programs are limited. Immunoadsorption (IA) enables selective antibody removal and is widely preferred, but the high cost of single-use IA columns restricts its routine use in low-resource settings. To address this challenge, some Indian centers have adopted structured column reuse under controlled protocols. This study was undertaken to evaluate the clinical safety, immunological efficacy, and practical feasibility of an IA-based desensitization protocol incorporating planned column reuse.

Aim: To evaluate the efficacy and safety of immunoadsorption-based desensitization using structured column reuse in ABO-incompatible transplantation.

Materials and methods: This was a retrospective study of 13 adult patients who underwent living donor ABO-incompatible kidney transplantation between June 2023 and May 2025. Desensitization involved IA with selective column reuse under validated sterile conditions. All patients received standardized immunosuppression, and isoagglutinin titers were serially monitored. Outcomes included titer reduction, graft function, antibody rebound, rejection episodes, and procedure-related complications. Statistical analysis was performed using standard methods, with p < 0.05 considered significant.

Results: Thirty-five IA sessions were conducted; 60% involved column reuse. Final IgG and IgM titers ≤1:8 were achieved in all patients prior to transplantation. Immediate graft function was seen in 12 patients. Mean serum creatinine at discharge was 1.07 mg/dL. No cases of antibody-mediated rejection, post-transplant isoagglutinin rebound, or reuse-related adverse events were observed. No patients required blood product transfusion or additional apheresis post-transplant. At three months, all patients had functioning grafts, with 100% patient and graft survival. The protocol demonstrated safety, efficacy, and feasibility within a cost-constrained setting.

Conclusion: Immunoadsorption with structured column reuse is an effective, safe, and practical desensitization strategy in ABO-incompatible transplantation, offering a viable approach in resource-limited healthcare systems.

## Introduction

Kidney transplantation is the preferred treatment for end-stage renal disease (ESRD), offering superior survival and quality of life compared to maintenance dialysis [1]. In high-income countries, this success is supported by robust deceased donor programs and centralized organ-sharing systems. By contrast, over 90% of renal transplants in India rely on living donors, mainly family members, making donor availability a persistent challenge [2,3]. Within this framework, ABO blood group incompatibility excludes up to one-third of otherwise suitable donor-recipient pairs, posing a major immunological barrier to transplant access [4].

ABO-incompatible kidney transplantation (ABOi-KT), once deemed an absolute contraindication, is now clinically feasible through structured desensitization protocols that combine immunosuppressive therapy with extracorporeal antibody removal [5-8]. When isoagglutinin titers are sufficiently reduced, outcomes can approximate those of ABO-compatible transplants, offering a critical solution for patients lacking compatible donors [9].

Among desensitization strategies, IA offers distinct advantages due to its selective removal of anti-A or anti-B isoagglutinins while preserving essential plasma components [10]. Most desensitization protocols integrate IA with anti-CD20 monoclonal antibody therapy and triple immunosuppression, aiming for pre-transplant titers of ≤1:8 as a benchmark for safe engraftment [4,6].

The availability of columns such as Glycosorb®, Adsopak®, and the newer SECORIM® has expanded the reach of IA-based protocols. These columns utilize immobilized carbohydrate antigens for targeted antibody removal. However, single-use IA columns remain prohibitively expensive in low- and middle-income countries (LMICs), where transplant costs are largely borne by patients [11,12]. This has prompted Indian centers to adopt cost-adaptive measures, including structured column reuse.

Although emerging data suggest that column reuse is both feasible and safe, large-scale evidence from academic public institutions is limited [13,14]. We therefore present our experience involving 13 consecutive ABO-incompatible living donor kidney transplants using an IA protocol with SECORIM® columns. The study evaluates antibody kinetics, safety, graft function, and short-term outcomes, with emphasis on column reuse as a cost-conscious strategy in the Indian healthcare context.

## Materials and methods

Study design and setting

This retrospective observational study was conducted at a tertiary transplant center in India between June 2023 and May 2025. It included 13 consecutive adult patients with end-stage renal disease (ESRD) who underwent living donor ABO-incompatible kidney transplantation following desensitization with immunoadsorption (IA). The study protocol was approved by the Institutional Ethics Committee (Ref. No.: SKCC/IEC/2025/07/PNO3) and conducted in accordance with the principles of the Declaration of Helsinki. The committee granted a waiver of written informed consent, as the study was retrospective in nature.

Inclusion criteria

Adults (≥18 years) with end-stage renal disease (ESRD) undergoing living-donor kidney transplantation, who have confirmed ABO incompatibility between donor and recipient, baseline anti-ABO isoagglutinin titers ≥1:32, and are eligible for immunoadsorption (IA)-based desensitization and immunosuppressive therapy.

Exclusion criteria

ABO-compatible donor-recipient pairs. Presence of active infection, malignancy, or uncontrolled systemic disease. Contraindications to immunoadsorption (IA) or immunosuppressive therapy.

Immunosuppressive protocol

All patients received a standardized immunosuppressive regimen. Rituximab (200 mg intravenous) was administered 14 to 21 days prior to transplantation. Mycophenolate mofetil was initiated 10 days before surgery at a dose of 500-750 mg twice or thrice daily, depending on patient tolerance. Tacrolimus was started seven days pre-transplant at an initial dose of 0.03 to 0.06 mg/kg/day, targeting trough levels of 8-10 ng/mL by the time of transplantation. Oral prednisolone (10-20 mg/day) was commenced seven days prior to and continued through the perioperative period. On the day before surgery, patients received intravenous methylprednisolone (500 mg).

Induction immunosuppression consisted of rabbit anti-thymocyte globulin (rATG) at a total dose of 3-4.5 mg/kg, administered in divided doses over three days. Maintenance immunosuppression included tacrolimus, mycophenolate mofetil, and corticosteroids, with dose modifications based on clinical and laboratory parameters. Early initiation of tacrolimus and mycophenolate mofetil may have contributed to isoagglutinin suppression, thereby complementing the effects of immunoadsorption.

Immunoadsorption protocol

Desensitization was performed using SECORIM®-ABO immunoadsorption columns (Vitrosorb AB, Sweden), which are designed for the selective removal of anti-A or anti-B isoagglutinins while preserving essential plasma components such as immunoglobulins, coagulation factors, and albumin. Each column contains 70 mL of polymethacrylate adsorption matrix functionalized with synthetic oligosaccharide antigens, housed in a polycarbonate case with polyethylene filter material and DEHP-free PVC tubing. The priming volume is 34 mL, and the column connects via standard male and female Luer Lock connectors.

Plasma separation was conducted using Fresenius 4008S hemodialysis machines fitted with P2 dry plasma filters. Separated plasma was passed through the SECORIM® column and returned to the patient. Plasma flow rates were maintained between 20 and 50 mL/min, with corresponding blood flow rates gradually increasing from 50 to 70 mL/min depending on patient hemodynamic stability. Each IA session processed approximately 2500-3000 mL of plasma (1.5-2 times the estimated plasma volume), with a cumulative average volume of ~8000 mL per patient. Anticoagulation was maintained using heparin according to institutional protocol.

Monitoring and titer assessment

Anti-ABO IgG and IgM titers were assessed before and after each IA session using standard tube agglutination. The target pre-transplant titer was ≤1:8, with sessions repeated as needed to achieve this. Post-transplant monitoring included daily serum creatinine, isoagglutinin titers, and clinical assessment for rejection.

Column reuse protocol and preservation

Although intended for single use, column reuse was implemented in 12 of 13 patients due to financial constraints. Reuse was selectively undertaken in individuals with persistently elevated isoagglutinin titers following the first immunoadsorption (IA) session. Column reuse in the same patient was performed with strict adherence to aseptic technique and institutional quality assurance protocols, as clinically required. After each IA session, the column was flushed with 1 L of normal saline containing 5000 units of unfractionated heparin to maintain patency and reduce the risk of thrombus formation.

The flushed column was then sealed, labelled with patient-specific identifiers, and stored at 4-8°C in a dedicated refrigerator. Columns were reused exclusively for the same patient and never shared between recipients. Prior to reuse, each column underwent visual inspection for discoloration, turbidity, or clot formation, and was discarded if any compromise was noted.

Data collection and statistical analysis

Baseline data collected included demographics, ABO pairing, HLA mismatch, dialysis duration, antibody titers, number of IA sessions, and transplant outcomes. Outcomes assessed were titer reduction, creatinine trends, immunologic stability, and adverse events. Statistical analysis was conducted using IBM Corp. Released 2020. IBM SPSS Statistics for Windows, Version 26. Armonk, NY: IBM Corp. Continuous variables were expressed as mean ± standard deviation or median; categorical variables as frequencies and percentages. The Wilcoxon signed-rank test was used to compare pre- and post-IA titers, and the paired t-test was applied to evaluate changes in serum creatinine. A p-value <0.05 was considered statistically significant.

## Results

Patient demographics and transplant details

Thirteen patients underwent ABO-incompatible living donor renal transplantation using SECORIM®-ABO (Vitrosorb) columns between June 2023 and May 2025. The cohort included 11 males and two females, with a mean age of 41.8 years (range: 21-61). Renal diagnoses included diabetic nephropathy (n=5), chronic glomerulonephritis (n=6), one with genetically confirmed atypical hemolytic uremic syndrome (aHUS), autosomal dominant polycystic kidney disease (n=2), and one biopsy-proven Alport syndrome.

Recipient blood groups were predominantly O positive (n=11), followed by A positive (n=2). Donor groups included A positive (n=7), B positive (n=5), and AB positive (n=1). The most common donor relationship was spousal (n=7), followed by mother (n=2), father (n=1), maternal aunt (n=1), paternal aunt (n=1), and sister-in-law (n=1).

Three patients (23%) received preemptive transplants; the rest were on maintenance hemodialysis, with a median vintage of seven months (range: 4-10). HLA matching ranged from 0/6 to 4/6, with four patients fully mismatched and three having 4/6 matches. All received uniform induction with rATG and maintenance immunosuppression comprising prednisolone, tacrolimus, and mycophenolate mofetil. All patients successfully underwent desensitization (Table 1).

**Table 1:** Recipient and donor characteristics. ADPKD: autosomal dominant polycystic kidney disease; aHUS: atypical hemolytic uremic syndrome; CGN: chronic glomerulonephritis; CKD 5D: chronic kidney disease stage 5 on dialysis; T2DM: type 2 diabetes mellitus; DN: diabetic nephropathy; HLA: human leukocyte antigen; IS: immunosuppression; MMF: mycophenolate mofetil; Pred: prednisolone; Tac: tacrolimus; rATG: rabbit anti-thymocyte globulin; +VE: positive.

Age (yrs)	Gender	Primary Diagnosis	Recipient Blood Group	Donor Blood Group	Donor Relation	HLA Match	Dialysis vintage (months)	Maintenance IS	Induction IS
46	M	T2DM/DN/CKD 5D	O +VE	A +VE	Wife	0 / 6	9	Pred/Tac/MMF	rATG
43	M	T2DM/DN/CKD 5D	O +VE	AB +VE	Wife	0 / 6	7	Pred/Tac/MMF	rATG
21	M	CGN/ CKD 5	A +VE	B +VE	Mother	4 / 6	PRE-EMPTIVE	Pred/Tac/MMF	rATG
37	M	ADPKD/CKD 5D	O +VE	A +VE	Paternal Aunt	4 / 6	6	Pred/Tac/MMF	rATG
61	M	T2DM/DN/CKD 5D	O +VE	A +VE	Wife	0 / 6	9	Pred/Tac/MMF	rATG
50	F	CGN (aHUS)	O +VE	A +VE	Husband	1 / 6	PRE-EMPTIVE	Pred/Tac/MMF	rATG
27	F	T2DM/DN/CKD 5D	O +VE	A +VE	Mother	4 / 6	4	Pred/Tac/MMF	rATG
36	M	CGN/Alport syndrome/CKD 5D	O +VE	A +VE	Wife	3 / 6	10	Pred/Tac/MMF	rATG
22	M	CGN/CKD 5D	O +VE	B +VE	Father	3 / 6	PRE-EMPTIVE	Pred/Tac/MMF	rATG
40	M	CGN/CKD 5D	A +VE	B +VE	Maternal Aunt	2 / 6	8	Pred/Tac/MMF	rATG
52	M	CGN/CKD 5D	O +VE	A +VE	Wife	0 / 6	7	Pred/Tac/MMF	rATG
52	M	T2DM/DN/CKD 5D	O+VE	B +VE	Wife	1 / 6	6	Pred/Tac/MMF	rATG
57	M	ADPKD/CKD 5D	O +VE	B +VE	Sister-In-Law	1 / 6	4	Pred/Tac/MMF	rATG

Baseline isoagglutinin titers, IA sessions, and column reuse

Baseline anti-ABO IgG titers ranged from 1:32 to 1:1024 and IgM from 1:16 to 1:512. Median baseline IgG was 1:256 (mode: 1:512 in five patients), and IgM was 1:64 (mode: 1:32 in four). One patient had IgG 1:1024; three had IgM 1:512.

Thirty-five IA sessions were conducted. Most patients (n=10) required 2-3 sessions; two required four, and one achieved desensitization with a single session despite an IgG titer of 1:512 and IgM of 1:256.

Column reuse was implemented in 12 of 13 patients. Of the 35 sessions, 60% used reused columns. Five patients underwent one reuse, five had two, and two required three reuse cycles. One patient completed desensitization without reuse. No mechanical issues, efficacy loss, or reuse-related complications occurred (Table 2).

**Table 2:** Anti-ABO antibody titers, immunoadsorption details, and admission creatinine levels in the study cohort. IS: immunosuppression; IA: immunoadsorption; IgG: immunoglobulin G; IgM: immunoglobulin M; Pre tx: pre-transplant; Creat: creatinine.

Baseline IgG Titer	Baseline IgM Titer	Post-Rituximab/IS titres (IgG/ IgM)	Total No. of IA Sessions	No. of Columns Used	R E U S e	Final Pre tx IgG Titer	Final Pre tx IgM Titer	Create Pre tx (mg/dL)
(1:128)	(1:32)	(1:64)/ (1: 16)	2	1	1	(1:8)	(1:2)	7.6
(1:512)	(1:512)	(1:256)/ (1:256)	4	1	3	(1:8)	(1:4)	8.6
(1:128)	(1:32)	(1:64)/ (1: 16)	2	1	1	(1:2)	0	6.4
(1:128)	(1:32)	(1:64)/ (1: 16)	3	2	1	(1:4)	(1:2)	7.9
(1:512)	(1:512)	(1:256)/ (1:256)	2	1	1	(1:8)	(1:8)	2.85
(1:512)	(1:512)	(1:256)/ (1:256)	3	1	2	(1:4)	(1:2)	8.01
(1:512)	(1:256)	(1:256)/ (1:128)	1	1	0	(1:8)	(1:4)	5.12
(1:512)	(1:256)	(1:256)/ (1:128)	3	1	2	(1:8)	(1:8)	9.05
(1: 32)	(1:16)	(1: 16)/ (1:8)	2	1	1	(1:8)	(1:4)	8.93
(1:64)	(1:32)	(1: 32)/ (1: 16)	3	1	2	(1:8)	(1:4)	8.33
(1:1024)	(1:256)	(1:512)/ (1:128)	3	1	2	(1:8)	(1:8)	7.34
(1:128)	(1:64)	(1:64)/ (1:32)	3	1	2	(1:8)	(1:8)	11.2
(1:256)	(1:64)	(1:128)/ (1:32)	4	1	3	(1:4)	(1:2)	11.71

Isoagglutinin titer reduction

A gradual decline in baseline anti-ABO IgG and IgM titers was observed following rituximab and initial immunosuppressive therapy, even before initiation of immunoadsorption. Subsequent IA sessions led to a substantial further reduction. All 13 patients ultimately achieved pre-transplant IgG and IgM titers ≤1:8, with one patient demonstrating complete IgM negativity. These findings underscore the combined efficacy of rituximab-based immunosuppression and SECORIM®-based immunoadsorption, even in patients with high initial isoagglutinin titers (Table 2).

The Wilcoxon signed-rank test demonstrated a statistically significant reduction in both IgG (Z = 0.0, p = 0.003) and IgM titers (Z = 0.0, p < 0.001) following IA (Table 3).

**Table 3:** Comparison of anti-ABO IgG and IgM titers before and after IA (Wilcoxon signed-rank test) IgG: Immunoglobulin G; IgM: Immunoglobulin M; IA: Immunoadsorption.

Titer type	Median pre-IA	Median post-IA	Z-value	p-value
IgG	1:256	≤1:64	0.0	0.003
IgM	1:64	≤1:8	0.0	<0.001

Graft function and early post-transplant outcomes

Admission creatinine ranged from 2.85 to 11.71 mg/dL. In preemptive transplants, it ranged from 6.4 to 8.01 mg/dL. All patients showed declining creatinine post-transplant, with discharge levels between 0.8 and 1.44 mg/dL (mean ~1.07 mg/dL), reflecting prompt graft function. Immediate graft function occurred in 12 of 13 patients. One preemptive patient had delayed graft function, which resolved conservatively.

The paired t-test confirmed a significant decline in serum creatinine from admission to discharge (t = 10.23, p < 0.001) (Table 4). 

**Table 4:** Comparison of serum creatinine pre- and post-transplant (paired t-test). IA: Immunoadsorption; SD: Standard deviation.

Time point	Mean ± SD (mg/dL)	t-value	p-value
Pre-transplant	8.02 ± 2.1	10.23	<0.001
At discharge	1.09 ± 0.2		

Post-transplant immunological and clinical monitoring

All patients maintained stable graft function during the early post-transplant period. No antibody-mediated rejection (ABMR) or rebound in anti-ABO titers was observed. No patient developed de novo donor-specific antibodies (DSAs). Tacrolimus levels remained therapeutic, and none required additional IA or plasmapheresis.

Graft survival and rejection events

At discharge, all patients were alive with functioning grafts, reflecting 100% patient and graft survival at three months. One patient had borderline T-cell-mediated rejection on protocol biopsy at two weeks, managed successfully with corticosteroids. No surgical, infectious, or immunologic readmissions occurred.

Procedural safety and infectious complications

IA was well tolerated, with no adverse events attributed to column use. There were no episodes of hypotension, allergic reactions, hemolysis, bleeding, thrombosis, or catheter-related issues. No patient required transfusion of blood products, fresh frozen plasma, or albumin during desensitization or post-transplantation. There were no cases of cytomegalovirus (CMV) reactivation, BK virus nephropathy, sepsis, or urinary tract infection (UTI) requiring hospitalization, affirming both procedural safety and effective immunoprophylaxis.

## Discussion

The emergence of ABO-incompatible kidney transplantation (ABOi-KT) as a standard-of-care option marks a major advance in renal replacement therapy, particularly in regions where deceased donor programs are underdeveloped and compatible donors are scarce [15,16]. The key to ABOi-KT success lies in achieving safe, effective desensitization [17]. Our study adds to the growing body of evidence by showing that immunoadsorption (IA), combined with structured column reuse, offers a clinically sound and economically feasible solution in resource-constrained settings.

IA's advantage lies in its selective removal of anti-A and anti-B isoagglutinins, preserving vital plasma proteins, clotting factors, and immunoglobulins [18-21]. While IgG antibodies are commonly monitored, IgM isoagglutinins, owing to their complement-fixing potential, also play a crucial role in early antibody-mediated injury [22]. Our results highlight effective titer reduction, with IA lowering both IgG and IgM to <1:8, even in patients with baseline IgG ≥1:1024 and IgM ≥1:512. These findings align with global experience from Rostaing et al. and Tyden et al. and Indian data from Jha et al., where titers reduced after using IA columns [23-25]. No patient experienced post-transplant antibody rebound or needed further IA, supporting the adequacy of a pre-transplant IA protocol alone, consistent with European protocols and findings from Rostaing et al. [23].

A decline in isoagglutinin titers was observed following administration of rituximab and early initiation of immunosuppressive therapy, even prior to the commencement of immunoadsorption. This pharmacologic immunosuppression likely exerted a synergistic effect, particularly in high-titer patients, consistent with prior studies demonstrating delayed suppression of anti-ABO IgG by calcineurin inhibitors and anti-metabolites.

The mean number of IA sessions in our cohort was 2.8, comparable to or lower than that reported by Tiwari et al. (3.2) and Rostaing et al. (2.6) [23,26]. This efficiency is likely reflected by the individualized, titer-based approach and effective reuse protocol. A study by Schiesser et al. and an Indian study by Mukherjee et al. similarly affirm that column reuse, when rigorously implemented, does not compromise desensitization efficacy [27,28]. A key strength of our protocol was structured reuse in 12 of 13 patients, with 60% of 35 sessions using reused columns, some up to three times, without loss of efficacy or safety. These findings mirror limited reports from Switzerland and Japan and substantiate Mukherjee et al.'s Indian experience [28]. In our context, reuse was not a compromise but a rational, safe response to cost pressures.

Desensitization yielded excellent early graft outcomes: mean creatinine fell from 8.02 mg/dL to 1.09 mg/dL at discharge, mirroring the trajectories reported by Rostaing et al. and Tiwari et al. in SECORIM®-based protocols. All patients maintained stable graft function [23,29]. There were no cases of antibody-mediated rejection (AMR), and only one instance of borderline T-cell-mediated rejection, which responded to corticosteroids. These outcomes are consistent with Rostaing et al. and reinforced by a meta-analysis by Genberg et al. showing IA-based protocols carry a lower AMR risk, likely due to more targeted antibody removal [23,30].

Importantly, no patient experienced bleeding or transfusion-related complications. IA preserves coagulation and avoids the need for plasma or albumin replacement. The limited number of sessions in our reuse-based approach may have further contributed to this favorable safety profile. Cost remains the major barrier to IA in India, where most healthcare costs are borne out of pocket. Despite being the preferred modality in the national consensus on ABOi-KT, its widespread adoption is restricted by expense. Our findings, along with those of Schiesser et al. and Mukherjee et al., suggest that structured reuse can make IA viable without compromising safety or efficacy, a highly relevant strategy for other low- and middle-income countries seeking sustainable transplant solutions [27,28].

All patients achieved pre-transplant titers ≤1:8, which is widely regarded as an acceptable target. While some centers aim for <1:4, accumulating evidence supports ≤1:8 as sufficient when post-transplant surveillance is robust [23]. Our protocol reflects this balanced approach, avoiding unnecessary overtreatment. Although most published reports focus on Glycosorb® or Adsopak®, our study supports SECORIM® as a valid IA platform. Mukherjee et al. have also shown favorable results with this column type [28]. Ultimately, our data reinforce that outcomes depend more on immunological assessment, protocol fidelity, and monitoring than on the specific column used.

Operationally, IA was well tolerated and logistically efficient. No patients had hypotension, hemolysis, catheter complications, or required blood products. IA required no albumin or plasma. The immunosuppressive regimen was well tolerated, with only one patient requiring intervention for borderline rejection. Our study has limitations, such as a small sample size, short follow-up, and lack of complement-binding assays (e.g., C1q, C3d). Formal cost-effectiveness modeling was not done. Still, consistency across clinical, immunologic, and safety outcomes suggests that structured reuse of IA columns is both practical and effective.

Future work should include multicenter prospective studies with longer follow-up, detailed immunologic profiling, and formal health-economic analyses. Such efforts are essential to validate the sustainability of reuse protocols and guide transplant policy in resource-limited regions where access to transplantation remains a pressing need.

## Conclusions

This study affirms that IA-based desensitization is a safe and effective strategy for ABO-incompatible kidney transplantation, including in high-titer patients. It further demonstrates that structured reuse of IA columns, when performed under sterile, validated conditions, provides a clinically sound and financially sustainable solution in resource-limited settings. SECORIM® columns were effective across a wide immunologic spectrum, with no adverse outcomes observed. In India’s cost-sensitive healthcare landscape, this approach offers a pragmatic path to broader transplant access. Future studies should translate these findings into a standardized, resource-adapted national protocol.
